# THE ASSOCIATION OF RESILIENCE AND SOCIAL NETWORKS WITH PAIN OUTCOMES AMONG OLDER ADULTS

**DOI:** 10.1093/geroni/igz038.1183

**Published:** 2019-11-08

**Authors:** Shirley Musich, Shaohung Wang, Luke Slindee, Sandra Kraemer, Charlotte Yeh

**Affiliations:** 1 Optum, Ann Arbor, Michigan, United States; 2 Optum, Eden Prairie, Minnesota, United States; 3 UnitedHealthcare, Minneapolis, Minnesota, United States; 4 AARP Services, Inc., Washington, District of Columbia, United States

## Abstract

Depression, stress and poor sleep are associated with increased pain among older adults with chronic pain. Positive resources may help buffer the impacts of negative attributes on pain. Our primary objective was to determine effects on pain outcomes (severity; interference) of positive resources (resilience; social networks) on negative attributes among older adults with pain. The sample (N=15,000) came from older adults≥65 years with AARP®Medicare Supplement and AARP®MedicareRx plans (insured by UnitedHealthcare Insurance Company) with diagnosed back pain, osteoarthritis and/or rheumatoid arthritis. Members received a survey assessing positive resources, negative attributes and outcomes of pain. Depression, stress, sleep, resilience, social networks, pain severity and interference were measured. Opioid and other medications were determined from claims. The population was propensity weighted to adjust for survey non-response; weighted to be generalizable to members with diagnosed pain. Multinomial logistic regression was used to determine associations of positive/negative attributes on pain. Among respondents (N=4,161; 29%), prevalence of pain severity and interference for no/mild, moderate and severe categories was 61%, 21% and 18% for severity and 67%, 16% and 17% for interference. In bivariate models adjusted for demographics/health status, negative attributes of depression, stress and poor sleep had stronger associations with pain severity and interference than moderating effects. In full multivariate models, the strongest associations with moderate and severe severity and interference remained depression, stress and sleep. Based on results, multidimensional pain management strategies should include management of depression, stress and poor sleep along with enhancement of positive resources and analgesics as needed for pain management.

